# The Potential Utility of Prebiotics to Modulate Alzheimer’s Disease: A Review of the Evidence

**DOI:** 10.3390/microorganisms9112310

**Published:** 2021-11-06

**Authors:** Jea Woo Kang, Angela M. Zivkovic

**Affiliations:** Department of Nutrition, University of California, Davis, CA 95616, USA; jwkkang@ucdavis.edu

**Keywords:** gut microbiome, Alzheimer’s disease, gut-brain axis, prebiotics

## Abstract

The gut microbiome has recently emerged as a critical modulator of brain function, with the so-called gut-brain axis having multiple links with a variety of neurodegenerative and mental health conditions, including Alzheimer’s Disease (AD). Various approaches for modulating the gut microbiome toward compositional and functional states that are consistent with improved cognitive health outcomes have been documented, including probiotics and prebiotics. While probiotics are live microorganisms that directly confer beneficial health effects, prebiotics are oligosaccharide and polysaccharide structures that can beneficially modulate the gut microbiome by enhancing the growth, survival, and/or function of gut microbes that in turn have beneficial effects on the human host. In this review, we discuss evidence showing the potential link between gut microbiome composition and AD onset or development, provide an overview of prebiotic types and their roles in altering gut microbial composition, discuss the effectiveness of prebiotics in regulating gut microbiome composition and microbially derived metabolites, and discuss the current evidence linking prebiotics with health outcomes related to AD in both animal models and human trials. Though there is a paucity of human clinical trials demonstrating the effectiveness of prebiotics in altering gut microbiome-mediated health outcomes in AD, current evidence highlights the potential of various prebiotic approaches for beneficially altering the gut microbiota or gut physiology by promoting the production of butyrate, indoles, and secondary bile acid profiles that further regulate gut immunity and mucosal homeostasis, which are associated with beneficial effects on the central immune system and brain functionality.

## 1. Introduction

Microbiota dysbiosis, characterized as the disproportional increase or decrease in abundance of certain bacterial strains, has been associated with multiple complications, including obesity [1], type 2 diabetes (T2DM) [2], and neurodegenerative diseases such as Alzheimer’s Disease (AD) [3]. AD is the most common neurodegenerative disease affecting about 5 million people in the U.S., and about 25 million people worldwide [4]. Only about 5–10% of AD patients present with early onset dementia directly linked to genetic mutations that are causal for AD development [5]. The vast majority of AD patients, on the other hand, develop neurodegenerative disease due to a combination of factors including but not limited to apolipoprotein E genotype [6,7], presence of metabolic syndrome and certain lifestyle factors [8], and, as recently revealed, microbiome composition [3]. AD is a neurodegenerative disease characterized by memory loss and a progressive loss of cognitive function involving the extracellular accumulation of pathogenic amyloid-β (Aβ) peptides that oligomerize and aggregate, forming plaques [9], and the intracellular accumulation of hyperphosphorylated tau proteins that form neurofibrillary tangles [10]. The causes for the formation of Aβ plaques and neurofibrillary tangles are not clear. However, chronic neuroinflammation and dysfunctional microglia have emerged as key drivers of these processes [11,12]. Notably, neuroinflammation has recently been found to be modulated by the gut microbiome via the gut-brain axis [13]. The links between microbiome composition and AD are intriguing and provide potential ways to ameliorate or even prevent AD progression through modifying the microbiome. This could be achieved via various ways, including fecal transplant and consumption of probiotics or prebiotics. Prebiotics are oligosaccharide molecules that are non-digestible to the human host, and which serve as substrates for microorganisms in the gut, and thus modulate the composition and/or function of gut microbes in a manner that is beneficial to the host [14,15]. In this review, we discuss the evidence linking gut microbiome composition and function with AD and its associated co-morbidities, provide an overview of prebiotic types and their effects, discuss evidence for the effectiveness of prebiotics in modulating gut microbiome composition and microbial metabolite production, and discuss the potential for prebiotics to induce a beneficial shift in the gut microbiome and modify health outcomes relevant for individuals with AD.

## 2. Links between Gut Microbiome Composition and AD and Associated Co-Morbidities

**Figure 1:**
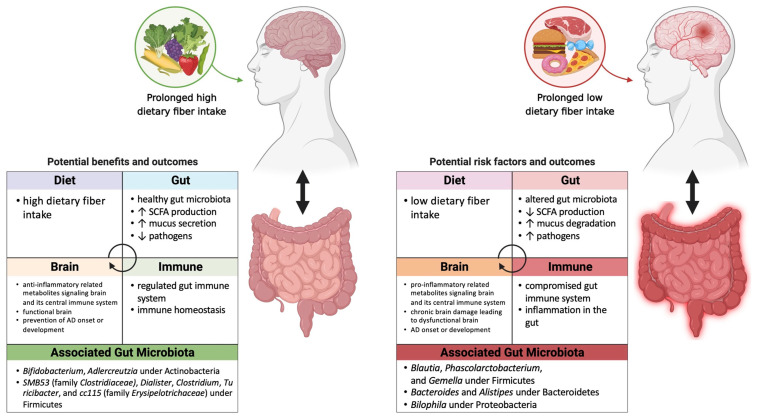
The potential association of prebiotic-gut-Alzheimer’s Disease (AD) in individuals with prolonged high vs. low fiber diet. The intake of dietary fiber may further influence gut health, immune system, and brain function. High dietary fiber intake may help maintain healthy gut microbiota, which is associated with increase in SCFA production, mucus secretion and decrease in pathogens. Healthy gut physiology leads to regulated gut immune system and immune homeostasis in which positively affects the brain. Anti-inflammatory metabolites signal the brain and its central immune system, which may potentially contribute to functional brain and prevention of AD onset or development. Bacterial genera that are shown to be less abundant in AD patients were *Bifidobacterium* and *Adlercreutzia* under Actinobacteria, *SMB53* (family *Clostridiaceae)*, *Dialister*, *Clostridium*, *Turicibacter*, and *cc115* (family *Erysipelotrichaceae*) under Firmicutes. Low dietary fiber intake may alter gut microbiota leading to dysbiosis in the gut, decrease in SCFA production, and increase in pathogens. Dysbiosis in the gut may cause compromised gut immune system and inflammation in the gut. Pro-inflammatory metabolites signal the brain and its central immune system and potentially bring chronic damage to the brain, which may result in dysfunctional brain, AD onset or development. Bacterial genera that are shown to be more abundant in AD patients were *Blautia*, *Phascolarctobacterium*, and *Gemella* under Firmicutes, *Bacteroides* and *Alistipes* under Bacteroidetes, *Bilophila* under Proteobacteria.

The importance of diet in modifying the gut microbiome has been emphasized through many intervention studies in humans and animal models. Studies have demonstrated that diet affects gut microbiota composition and diversity [16,17,18,19,20,21,22,23,24,25]. Diet composition and duration of intervention are the two most relevant diet-related factors in shaping the gut microbiome. The most well-studied dietary interventions thus far have involved the comparison of high-fat or Western diets enriched in animal-derived foods vs. lower-fat or plant-based diets (Figure 1). From animal studies to human studies the diversity and proportion of microbes have been found to be consistently altered by diets depleted vs. enriched in plant substrate. Specifically, diets depleted in non-digestible fiber and enriched in protein and fat have been consistently linked with an increase in protein- and fat-degrading bacteria belonging to the phyla Firmicutes, Proteobacteria, and Deferribacteres, and a decrease in Bacteroidetes and butyrate-producing species, which are generally known to be beneficial for human health [26,27,28,29,30]. Conversely, fiber-enriched diets are typically associated with increases in the abundance of species in the phylum Bacteroidetes, the genus *Prevotella*, and *Bifidobacterium* spp. [31,32,33,34,35]. These changes in gut microbiota composition are closely associated with host health and disease. The health effect is not only attributed to the enrichment of beneficial gut microbes but to the production of secondary metabolites such as short chain fatty acids (SCFAs) from the degradation of non-digestible carbohydrates by specific fiber-fermenting taxa [36,37,38]. The presence of these taxa is associated with protection from AD, and a number of associated co-morbidities including T2DM and cardiovascular disease (CVD). In the next several paragraphs we review the evidence linking gut microbiome alterations to AD, as well as associated co-morbidities.

Studies have shown a connection between the composition and diversity of gut microbes and AD (Figure 1) [3,39,40]. In a recent study a reduction in overall gut microbiome richness as well as decreases in *Bifidobacterium* and *Adlercreutzia* under Actinobacteria, *SMB53* (family *Clostridiaceae)*, *Dialister*, *Clostridium*, *Turicibacter*, and *cc115* (family *Erysipelotrichaceae*) under Firmicutes were observed in AD participants [3]. On the other hand, *Blautia*, *Phascolarctobacterium*, and *Gemella* under Firmicutes, *Bacteroides* and *Alistipes* under Bacteroidetes, and *Bilophila* under Proteobacteria were increased in AD patients [3]. In addition, 13 genera were associated with cerebrospinal fluid (CSF) biomarkers of AD [3], showing that gut microbiome composition or diversity may contribute to AD development. Firmicutes and Bacteroidetes are two dominant phyla in the human gut [41] and it has been observed that the Firmicutes/Bacteroidetes ratio is associated with obesity, gut dysbiosis, and a number of diseases including diabetes and CVD. However, the use of this ratio as an assessment of the health state of the gut microbiota is controversial, as contradictory results have been reported [3,39,42,43,44,45]. Gut microbiota composition assessment metrics that are based on measurements at the phylum level are unlikely to be useful since individual genera and species, even strains, under a particular phylum can play opposite roles in overall gut health, taking on different metabolic roles, producing different metabolites, and interacting with other microbes in the gut in different ways such that the overall effect of all individual species of that phylum is complex (Figure 1).

The onset and progression of AD has been linked directly to neurodegenerative processes secondary to the deposition of Aβ plaques and aggregation of hyperphosphorylated tau tangles [46]. Recently, the pathogenesis of AD has been hypothesized further to be triggered by amyloid fibers of bacterial origin, which induce a proinflammatory response [47]. A recent study found that amyloid-positive cognitively impaired patients had higher *Escherichia*/*Shigella* and lower *Eubacterium rectale* and *Bacteroides fragilis* abundances compared to amyloid-negative cognitively normal controls, and these compositional changes were correlated with an increased production of pro-inflammatory cytokines and a reduction of anti-inflammatory cytokines [48]. In a cross-sectional study in Australian women consumption of a “junk food” (high sugar, high fat) diet was highly associated with Aβ deposition, whereas consumption of the Mediterranean diet was associated with higher cognitive scores than other diet groups [49]. Interestingly, in a small study participants with mild cognitive impairment consuming a modified Mediterranean-ketogenic diet consisting of less than 20 g/d of carbohydrate were found to have higher abundances of *Enterobacteriaceae*, *Akkermansia*, *Slackia*, *Christensenellaceae* and *Erysipelotrichaceae* and lower abundances of saccharolytic *Bifidobacterium* and *Lachnobacterium* compared to cognitively normal participants [50]. In a follow-up study the low-carbohydrate modified Mediterranean-ketogenic was found to have a potential beneficial effect in AD patients in preventing memory decline [51]. However, these studies were conducted in small cohorts (e.g., 17 individuals, 11 MCI patients and 6 controls), thus the effects of low-carbohydrate, low-fiber diets, even in the context of high monounsaturated and polyunsaturated vs. saturated fat ratios such as those seen in the Mediterranean diet, need to be further investigated in larger trials.

T2DM and AD have been known to share several pathophysiological features including hyperglycemia leading to increased Aβ production, and impaired glucose transport and subsequent glucose metabolism [52]. A new potential AD biomarker, S100B, has been investigated to learn the common pathophysiology of these diseases [53]. A cross-sectional study conducted with 100 South Indian AD patients showed that elevated levels of S100B protein in serum were significantly associated with clinical dementia rating scores compared to healthy controls [54]. Serum S100B protein levels in T2DM patients were also shown to be positively correlated with cognitive function [55]. In patients with clinically diagnosed T2DM a high-fiber diet composed of whole grains and prebiotics promoted strain specific growth of acetate and butyrate producing bacteria *Faecalibacterium prausnitzii*, Lachnospiraceae bacterium, and *Bifidobacterium pseudocatenulatum* [56]. The treatment group had improved levels of hemoglobin A1c, as well as increased glucagon-like peptide-1 production compared to the control group [56]. These results suggest the high-fiber diet induced gut microbial alteration is correlated with improvement of blood glucose regulation in T2DM patients. These findings have important implications for the management of AD due to the high rates of T2DM comorbidity in AD patients.

In addition to a link with T2DM, CVD has also been linked with AD [57,58]. The occlusion of blood vessels that support the deep brain result in silent brain infarcts [59]. This type of infarct is shown to be associated with lower cognitive function related to attention, memory, and language [60]. CVD may directly affect poor blood flow to the brain causing cerebrovascular disease [61]. Meta-analyses of prospective cohort studies exploring the association of coronary heart disease with dementia or cognitive impairment found that coronary heart disease is associated with an increased risk of dementia or cognitive impairment [62,63]. It is well-established that as much as 80% of the risk for CVD is attributable to diet and lifestyle factors [64,65,66]. Many human studies have demonstrated an inverse association between the consumption of dietary fiber and the incidence of CVD [67,68,69,70,71]. Patients with primary hypertension showed a high frequency of opportunistic pathogens such as *Klebsiella* spp., *Streptococcus* spp., and *Parabacteroides merdae*, whereas *Roseburia* spp. and *F. prausnitzii* which are SCFA-producers were abundant in healthy individuals [72]. Another study found that total and LDL-cholesterol levels were lowered after the consumption of flaxseed fiber [73]. However, although the consumption of maize-derived whole grain cereal led to increases in bifidobacteria, no significant changes were observed in serum lipids [74]. Further studies examining the role of dietary fiber and specific increases or decreases of gut microbes as well as their metabolites on CVD endpoints are needed.

Taken together, the overall findings from the published literature suggest that modifying gut microbial composition and diversity toward a profile associated with healthy individuals consuming healthy diets may help attenuate AD progression. Diets and prebiotic approaches that aim to increase beneficial bacterial species that have been found to be depleted in AD patients such as *Bifidobacterium* spp., and approaches that aim to decrease the abundance of deleterious bacterial species such as *Bilophila* may be beneficial for the prevention of AD (Figure 1).

## 3. Overview of Prebiotic Types and Their Roles in Modifying Gut Microbiota

Dietary fibers, which are somewhat difficult to define, can be classified according to their solubility. Insoluble fiber, which does not dissolve in water, passes through the digestive tract providing bulking by absorbing water. Soluble fiber, on the other hand, dissolves in water and is mostly fermented by commensal bacteria residing in the colon and contributing to satiety [75,76]. Although this general categorization of fibers according to their solubility may be useful, insoluble fibers are fermented to a certain degree and some soluble fibers may be non-viscous. Recently, the classification of fiber according to functionality is gaining attention. The functionality depends on the structure and fermentability of the specific dietary fiber. Thus, types of dietary fiber and subsequent gut microbial composition, diversity, and richness changes are highly intriguing areas for further research. It is especially relevant to patients with AD given that particular dietary fibers may modify the gut microbiome in a beneficial direction, increasing the levels of metabolites that improve cognitive function and attenuate neurotoxicity [77]. Here, we have listed a number of dietary fibers with known impacts on enrichment of certain gut microbes, suggesting their potential as prebiotic supplements for AD patients (Table 1).

Cellulose and hemicellulose are major water-insoluble, non-starch polysaccharides found in plant cell walls. Cellulose degradation is known to be conducted by *Ruminococcus* spp. and *Bacteroides* spp. producing SCFAs as a byproduct [78,79,80]. Some species of gut microbes, including *Butyrivibrio* spp. *Clostridium* spp. and *Bacteroides* spp. are observed to break down hemicellulose [81]. Lignin is also a water-insoluble, non-starch polysaccharide that constitutes plant cell walls together with cellulose and hemicellulose, however its interaction with gut microbes is not well-documented. One study has shown that lignin supports the prolonged survival of bifidobacteria in an in vitro condition [82]. Resistant starch, another type of dietary fiber that is water-insoluble is a starch polysaccharide which is not degradable by the α-amylase enzyme of the host. Resistant starch was shown to increase the ratio of Firmicutes to Bacteroidetes [92]. At the genus level, *Bifidobacterium* and *Ruminococcus* have been identified to relatively thrive when exposed to resistant starch [83].

Fructan is a polymer of five carbon membered ring fructose molecules, which consists of several different types depending on the chemical bond. Fructo-oligosaccharide (FOS) and inulin are major forms of fructan considered as dietary fibers that are capable of being fermented by multiple members of the gut microbiota community [93]. FOS is a short chain oligosaccharide of fructose linked by β (2→1) glycosidic bonds. Inulin is a heterogeneous polysaccharide with β (2→1) linkage and terminal glucose. These fructan molecules have a bifidogenic effect that enhances the relative abundance of *Bifidobacterium* spp. in the host gut [84,85,86,94]. Similarly, galacto-oligosaccharide (GOS) is a short chain polymer of mainly galactose linked with a β (1→4) bond and terminal glucose [95]. FOS and GOS are commercially used to produce infant formula to mimic the properties of human milk [96]. These oligosaccharides are important nutrients to develop the gut microbiome of infants leading to colonization of beneficial bifidobacteria [97,98]. The promotion of these gut microbiota in infants decreases the niche for pathogenic bacteria and helps to enhance gut barrier function [87,99,100,101]. FOS supplementation in chronically stressed mice was demonstrated to prevent intestinal barrier impairment and neuroinflammation along with improved depression-like behavior and significant changes in the abundance of *Lactobacillus reuteri* [102]. FOS from *Morinda officinalis* were also tested in rats with AD-like symptoms and mice with inflammatory bowel disease showing the potential of FOS as a prebiotic that improved gut barrier integrity, alleviated neuronal degradation, downregulated AD markers, and maintained the diversity and stability of the gut microbiome of the host [103].

Beta-glucan is a polysaccharide that contains β-D-glucose linked by glycosidic bonds. A linear, non-branched β-glucan mostly found in the bran of cereals such as oats and barley is water-soluble and consists of β-D-glucose with (1→3), (1→4)-linkage [104]. This physicochemical property of β-glucan results in increased viscosity and a thickening effect on feces, and it provides beneficial, saccharolytic gut microbes with fermentable substrate to consume [105,106,107]. Consumption of high molecular weight β-glucan increased the proportion of *Bacteroides* and *Prevotella* [88]. Supplementation of either whole grain oats or oat bran elevated the production of SCFAs and produced a bifidogenic effect [89].

Pectin is a water-soluble dietary fiber mainly found in the skin of apples. Pectin is a component of the primary cell wall and middle lamella which contribute to adherence of adjacent plant cells. The structure of pectin is very complex and the pectic polysaccharides are abundant in galacturonic acids. Homogalacturonan is a polymer of galacturonic acid bonded with α-1,4-linkage and the types of pectin may vary according to its side chain sugars [108]. These complex pectins are known to be degraded by gut microbiota whose diversity is found to be preserved by pectin in ulcerative colitis patients [109]. Pectins derived from apples were found to be utilized by beneficial colonic bacteria including *Bifidobacterium*, *Lactobacillus*, *Enterococcus,* suggesting a prebiotic capacity of pectin [90].

Gums are commonly found in food thickeners because of their capability of gel formation and emulsion stabilization. Particularly, gum arabic is well determined for its solubility in water, becoming viscous depending on its concentration. Gum arabic is a complex heteropolysaccharide mainly containing 1,3-linked β-D-galactose units with 1,6-linked β-D-galactose side chains attached to rhamnose, glucuronic acid and arabinose residues [110,111]. It is accessible to the gut microbes having a potential to increase probiotic bacteria in the human gut. At a dose of 10 g for 4 weeks gum arabic resulted in significantly higher numbers of *Bifidobacterium*, *Lactobacillus*, and *Bacteroides* spp. in a human clinical trial [91].

The structural complexity of dietary fibers and the associated diversity of gut microbes that consume them require further research. It is important to determine the utilization of specific fibers by distinct microbiota and to demonstrate which structural traits and/or components of these fibers affect cognitive function via altering the gut microbiome in future studies.

## 4. Effectiveness of Prebiotics in Modulating Gut Microbiome Composition and Microbial Metabolite Production

**Figure 2:**
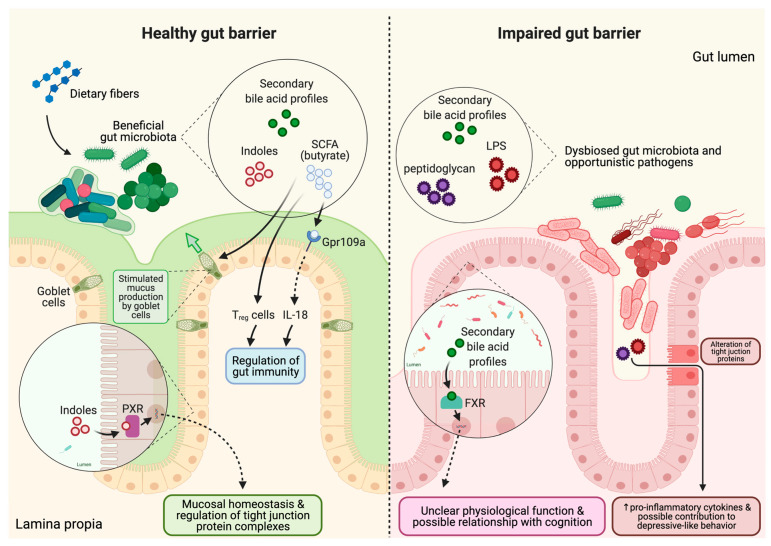
Gut barrier integrity changes and differences in signaling molecules in healthy vs. unhealthy gut. In healthy gut barrier, dietary fiber from diet is digested by the beneficial gut microbiota which produces secondary metabolites such as SCFA (butyrate), indoles, and secondary bile acid profiles. Butyrate is known to use Gpr109a as a receptor expressed in the enterocyte which produces IL-18, or it may directly affect T regulatory (T_reg_) cells. IL-18 and T_reg_ cells can both regulate gut immunity. SCFA also stimulates mucus production by goblet cells for healthy mucosal barrier. Indoles are ligands for pregnane X receptor (PXR) acting as transcription factor in sustaining mucosal homeostasis and regulation of tight junction complexes. Secondary bile acid profiles are ligands for farnesoid X receptor (FXR) and can be found in both healthy and unhealthy gut. The physiological roles of secondary bile acid profiles are unclear and may have possible relationship with cognition. In impaired gut barrier, gut microbiota are dysbiosed and byproducts such as peptidoglycan and LPS are released from opportunistic pathogens. The mucosal barriers are attenuated which provides more close contact of pathogens near enterocytes altering tight junction proteins. The peptidoglycan and LPS may pass through compromised tight junction increasing pro-inflammatory cytokines and possibly contributing to depressive-like behavior.

The overall impact of the gut microbiome on the production of microbial metabolites and gut barrier function is summarized in Figure 2.

The fermentation of dietary fiber or prebiotics by gut microbiota and the major metabolites from that process have been elucidated in many studies [112,113,114,115]. Particularly, butyrate is the preferred energy source of apical colonocytes [116]. Furthermore, SCFA lower the pH of the gut, suppressing the growth of pathogens [117], mediate gut immune regulation [118], and influence gut motility [119]. Thus, SCFAs act as signaling molecules that induce downstream pathways modulating the physiology, immunity, and metabolism of enterocytes. Gpr109a is a type of G protein-coupled receptor specifically activated by butyrate and is expressed in enterocytes, immune cells, and even in microglia [120,121,122]. Butyrate binding to the gpr109a receptor triggers several cellular signaling pathways (Figure 2) including those involving the colonic epithelium, macrophages, and dendritic cells. For example, Gpr109a signaling is known to promote anti-inflammatory properties by inducing IL-18 and IL-10 production, which induces differentiation of naïve T cells to T regulatory cells, thus supporting overall gut immunity by preventing colonic inflammation [123].

Neurotransmitters are another class of signaling molecule that plays an important role in the gut-brain axis. Serotonin, for example, is known to be mostly released from epithelial enterochromaffin cells [124,125]. The gut microbiota play a key role in promoting serotonin synthesis by host enterochromaffin cells. SCFA or secondary bile acids produced by gut microbes mediate serotonin production by enterochromaffin cells, which can further affect gut motility via the enteric nerve and brain serotonergic systems [126,127]. These findings suggest that certain prebiotic supplements, which stimulate the production of SCFA and secondary bile acids by specific microbes, can improve neurological function and behavior via upregulation of serotonin [128]. Another interesting neurotransmitter that connects gut and brain function is Gamma-aminobutyric acid (GABA). GABA is a crucial inhibitory neurotransmitter in the central nervous system and its alteration in GABAergic mechanisms is related to central nervous system disorders [129]. A recent study demonstrated the link between the gut microbiome (*Bacteroides* spp.) and GABA production, a response negatively correlated with depression [130]. Fecal microbiota from healthy control and schizophrenia patients were compared and each were transplanted to germ-free mice. Gut microbial dysbiosis shown in schizophrenia was related to changes in the GABA cycle which, in turn, may affect neurobehavioral status such as schizophrenia-relevant behaviors [131]. The production of neurotransmitters, particularly serotonin and GABA was distinctly linked with *Bifidobacterium* and *Lactobacillus* genera [132]. These findings highlight the potential role of prebiotics that promote the composition of these specific microbes, because their presence has been linked with decreased dysbiosis in the gut and the production of functional neurotransmitters, which may contribute to enhancing enteric health and attenuating AD-related neurobehavioral disorders.

In addition to neurotransmitters, prebiotics may also play an important role in regulating cytokine expression. Soluble fiber (pectin) treatment in mice resulted in faster recovery from endotoxin-induced sickness behaviors along with changes in the concentrations of cytokines, including IL-1RA, IL-4, IL-1β and TNF-α in the brain [133]. The pectin-supplemented mice also had increased concentrations of cecal acetate, propionate, and butyrate as a byproduct of pectin fermentation, which was associated with increased gastrointestinal IL-4 [133]. These findings suggest that soluble fiber not only affects the gastrointestinal tract and peripheral immune system but also neuroimmune system function. In another study in adult and aged mice a high fiber diet with inulin led to increased levels of cecal SCFA production including butyrate and acetate [134]. A reduction in inflammatory infiltrate was observed in the aged mice on the high fiber diet, and researchers specifically showed that sodium butyrate had anti-inflammatory effects on microglial profile, lowering inflammatory gene expressions [134]. These data suggest that butyrate produced from prebiotic fermentation may be a potent modulator of gut immune function and directly linked to microglial function in the brain.

Gut microbiota derived metabolites such as SCFA and indole are critical for sustaining intestinal barrier function (Figure 2). Acetate and butyrate, for example, improve goblet cell differentiation and stimulate mucus production by goblet cells to maintain healthy mucosal barrier [135]. Mice fed a low-fiber Western style diet were found to have a defect in mucin production, which was prevented by supplementation with a synbiotic of *Bifidobacterium longum* and inulin [136], suggesting that when SCFA-producing microbes are present in the gut along with a preferred substrate, the net effect is enhanced mucosal barrier function. In addition to a decrease in fiber-fermenting microbes and thus SCFA production, a diet deficient in fiber can also promote the enrichment of mucus-degrading gut microbes such as *Akkermansia muciniphila* [137]. *Bifidobacterium bifidum,* which has the ability degrade mucin [138] may protect thinning of the mucus layer by inhibiting *Akkermansia muciniphila,* as was shown in mice with omeprazole-induced small intestine injury [139]. Paradoxically, the presence of *Akkermansia muciniphila* has been linked with beneficial health effects [140,141,142,143], as well as negative health effects in individuals with certain health conditions [144,145]. The roles of specific microbes and their metabolites in the maintenance vs. degradation of the mucosal barrier are context-specific and require further study. Prebiotics may be a useful strategy to prevent mucus degradation by supporting the growth of SCFA-producing microbes and thus increasing mucin production, as well as sustaining the homeostasis of mucolytic vs. non-mucolytic bacteria in the gut.

Butyrate is known to regulate the expression of tight junction protein complexes [146]. Sodium butyrate was shown to increase Claudin-1 expression and induced redistribution of ZO-1 and Occludin in vitro [147]. Butyrate treatment accelerated the assembly of tight junctions by reorganizing the tight junction proteins in a Caco-2 cell monolayer model [148]. No studies have demonstrated a direct link between butyrate derived from the gut on tight junctions supporting endothelial cells that form the blood–brain barrier. However, these findings of a beneficial effect of butyrate on barrier function in the gut epithelium raises the question of whether a similar benefit may also be found in endothelial cells. A link between butyrate and brain function has been suggested. Bourassa et al. hypothesized that butyrate could be used as an important alternative energy substrate in the Alzheimer’s brain where glucose utilization has been found to be reduced [149,150,151].

Indoles are a class of molecules produced by gut microbes that have the potential to affect gut and brain function. In a germ-free mouse model, oral administration of indole led to up-regulation of tight and adherens junction-associated molecules in the epithelial cells of the colon [152]. Indole 3-propionic acid acts as a ligand for pregnane X receptor and increased expression of junctional protein-coding mRNAs while decreasing TNF-α in a mouse model [153]. The effect of indole 3-propionic acid was also tested in the Caco-2/HT29 coculture model and showed an increase in tight junction proteins, mucins, and goblet cell secretion products [154]. However, the role of indole and its derivatives is controversial in terms of the gut-brain axis [155,156]. Studies have demonstrated potent neuroprotective properties of indoles, which cross the blood–brain barrier and protect the brain from oxidative stress [157] as well as prevent electron leakage from neuronal mitochondria [158,159]. However, other studies report excessive production of indole by gut microbes may negatively affect emotional behavior in rats due to the neurodepressive properties of oxidized derivatives of indole, oxindole and isatin [160]. Indoxyl sulphate, an oxidized and sulphated form of indole produced from the liver, may reduce the efflux of neurotransmitters through the organic anion transporter 3, causing accumulation of metabolites [161,162]. Thus, the effects of indoles on gut barrier and brain function require further study, as the variety of indole metabolites produced by the gut microbes and their co-metabolism by the host generate a complex suite of molecules with differential effects.

Bile acids are a category of metabolite that is modulated by gut microbial metabolism, and which may have effects on the gut-brain axis. Bile acids are produced in hepatocytes and play a critical role in fat digestion and absorption. Most (95%) bile acids are recycled back to the liver via enterohepatic recirculation after reaching the terminal ileum. However, bile acids that are not recycled are excreted in feces or may be metabolized by the colonic microbiota, forming secondary bile acids via a series of microbial enzyme activities including deconjugation and 7α-dehydroxylation [163]. Thus, secondary bile acids are gut microbe-derived metabolites that may further regulate bile acid signaling of the host, affecting the activation of the enteroendocrine bile acid receptor, farnesoid X receptor (Figure 2) [164]. Several papers have shown a connection between bile acid metabolism and AD. In AD patients, significantly lower serum concentrations of a primary bile acid (cholic acid) and increased secondary bile acid (deoxycholic acid) were observed compared to cognitively normal older adults [165]. Increased deoxycholic acid to cholic acid ratio is known to be strongly associated with cognitive decline [166]. The ratio of primary to secondary bile acids was positively correlated with the abundance of *Bifidobacterium* in a human clinical trial [167]. Recently, alteration in bile acid profiles was shown to have an association with cognitive decline and AD-related genetic variants [165].

There are likely hundreds if not thousands of microbially produced molecules that likely play important roles in host health. Among these, butyrate, indole, and bile acids, are to date, the most well-studied, and their roles in gut health, brain function, and specific roles in the pathophysiology of AD, are starting to emerge. As we gain knowledge on both short-term and long-term effects of diet on the brain mediated by the gut microbiome, it will be important to establish a dossier of evidence of benefit of specific prebiotics for the pathophysiology of AD. In the following section, we discuss potential prebiotic approaches to supplement AD patients.

## 5. Current Evidence for Effectiveness of Prebiotics in AD Animal Models and Human Trials

The effectiveness of prebiotics for the treatment of AD will ultimately need to be evaluated on the basis of their ability to either improve or prevent cognitive decline. However, other symptoms of AD related to behavioral and emotional changes are also viable targets of prebiotic intervention studies in AD patients. The current literature showing the potential effects of prebiotics on cognitive function in both animal models and human studies mainly focuses on the effects of fructans, both in the form of oligosaccharides and inulin, β-glucan from yeast or the bran of cereals, plant polysaccharides, and polysaccharides synthesized from sugars. This evidence is summarized below.

### 5.1. Animal Models

Animal models have been used in several studies to evaluate the effect of prebiotics on AD, particularly mice due to their reliability on intervention and ease of sampling. In this section, animal studies on administration of prebiotics that led to improvement in AD associated brain disorders are summarized. Bimuno-GOS intake in pregnant mice affected the offspring’s exploratory behavior and brain gene expression as well as reducing anxiety [168]. Additionally, fecal butyrate and propionate levels were increased after Bimuno-GOS supplementation in postnatal mice [168]. In another study, behavioral testing was performed on mice from the least stressful (three-chamber test) to the most stressful (forced swim test) for 5 weeks during a 10-week prebiotic administration period including lead-in and lead-out periods [169]. The prebiotic treatment with a FOS+GOS combination resulted in a reduction of stress-related (depression and anxiety) behaviors, and reversed chronic stress (elevations in corticosterone and proinflammatory cytokine levels) in the supplemented mice compared to the control mice with no prebiotic treatment [169]. In a rat model exhibiting oxidative stress, mitochondrial dysfunction, and cognitive decline in the brain induced by high fat diet-induced obesity these outcomes were improved and cognitive function was restored by 12-week supplementation of either prebiotic (xylo-oligosaccharide), probiotic (*Lactobacillus paracasei* HII01), or combined treatment with similar efficacy [170]. The effectiveness of mannan-oligosaccharide was tested in a 5xFamilial AD transgenic mouse model [171]. The treatment with mannan-oligosaccharide reduced Aβ accumulation in the brain and suppressed neuroinflammatory responses [171]. Mannan-oligosaccharide not only improved cognitive and behavioral disorders, but also gut barrier integrity by reshaping the composition of gut microbiota, specifically increases in the relative abundances of *Lactobacillus* and decreases in *Helicobacter* [171]. Importantly, the observed changes in gut microbiota composition and butyrate production were negatively correlated with oxidative stress in the brain and behavioral deficits [171].

### 5.2. Human Trials

Studies on the effects of prebiotic supplementation directly on cognitive and behavioral outcomes in Alzheimer’s patients are currently lacking. However, a few human intervention studies were conducted to test the effectiveness of certain prebiotics alone or with probiotics on improving symptoms associated with AD such as behavioral, mood, memory, anxiety, and cognitive disorders.

Fructan and GOS-based prebiotics show promising and consistent results in clinical trials in decreasing anxiety and improving cognitive and behavioral outcomes. The prebiotic Bimuno-GOS improved antisocial behaviors in autistic children [172]. Trans-GOS stimulated bifidobacteria in the gut of irritable bowel syndrome patients and lowered anxiety [173]. Short chain FOS enhanced fecal bifidobacteria and reduced anxiety scores [174]. Inulin in healthy participants resulted in better recognition and improved recall [175]. In obese patients adhering to calorie restrictions for 3 months supplementation with 16 g/d of inulin had moderate impact on mood and cognition, with responders who experienced an increase in *Coprococcus* and *Bifidobacterium* having stronger benefits than non-responders [176]. Importantly, in most of these intervention studies, subjects supplemented with fructan or GOS prebiotics showed increases in bifidobacteria in general along with improvement in their symptoms. Many studies have already reported the connection between the increase in bifidobacteria and beneficial health outcomes (Table 1). Indeed, the growth of bifidobacteria is selectively stimulated by fructans [177]. The increase in *Bifidobacterium longum* 1714 strain in healthy mice showed stress resistance and pro-cognitive effects [178,179]. The same *Bifidobacterium* strain from this preclinical study displayed association with reduction in stress and improvement in memory in healthy volunteers [180]. The results from these studies suggest a strong connection between prebiotics, the gut microbiome, particularly bifidobacteria, and brain function.

Other studies provide supporting evidence that prebiotics modulate brain function in a manner that would be consistent with desired improvements in symptoms of AD but were not necessarily linked with or did not examine gut microbiome composition. Beta-glucans from yeasts, plants or cereals have been shown to have beneficial health effects on the profile of mood state in healthy individuals [181,182]. Plant polysaccharides, which mainly consist of non-starch polysaccharides found in foods were shown to have effect on healthy adults, improving their recognition and memory performance [183,184]. Polydextrose, which is a synthesized prebiotic, was supplemented in healthy females and showed moderate improvement in cognition as well as significant change in abundance of *Ruminiclostridium 5* compared to the placebo group [185]. Other studies have found 30–60 mL of lactulose for 3 months improved cognitive function and health-related quality of life in patients with minimal hepatic encephalopathy [186].

## 6. Concluding Remarks

Although human clinical studies examining the effects of specific prebiotics on gut microbiome-mediated cognitive health outcomes in AD patients are lacking, there is mounting evidence that prebiotics have the potential to be a viable approach for ameliorating symptoms associated with AD. Promoting the growth and activity of beneficial, SCFA-producing microbes such as bifidobacteria is emerging as a clear therapeutic target for improving gut barrier function, decreasing inflammation, and improving cognitive and behavioral outcomes. A variety of prebiotic types, particularly fructans, have been found to be effective in modulating gut microbiome composition and microbial metabolite production, and modifying health outcomes relevant for individuals with AD. More research is needed to determine which prebiotics, at what dosages, and in which context (e.g., on what dietary background, in combination with specific probiotics, at what frequency, etc.) are the most effective for not only decreasing AD-associated symptoms such as anxiety and depression, but also potentially improving cognition or preventing the loss of cognitive function in individuals at risk for AD. Further mechanistic research to determine how changes in the gut microbiome related to prebiotic supplementation alter neuroinflammatory signaling are also needed so that targeted, effective, potentially personalized therapies can be developed to treat and prevent the progression of neurodegenerative processes in AD.

## Figures and Tables

**Table 1:** Types of dietary fibers associated with the growth of certain gut microbiota.

Fiber Types	Main Features	Natural/Food Sources	Associated Gut Microbiota	References
Cellulose	Bulking	Plant cell wall	*Ruminococcus* spp. *Bacteroides* spp.	[78,79,80]
Hemicellulose	Bulking	Plant cell wall	*Butyrivibrio* spp. *Clostridium* spp. *Bacteroides* spp.	[81]
Lignin	Bulking	Plant cell wall	*Bifidobacterium* spp.	[82]
Resistant Starch	Fermentable	Seeds and unprocessed whole grains	*Bifidobacterium* spp. *Ruminococcus* spp.	[83]
Fructan				
Fructo-oligosaccharide (FOS)	Fermentable	Jerusalem artichoke, chicory, and the Blue Agave	*Bifidobacterium* spp.	[84]
Inulin	Fermentable	Wheat, bananas, asparagus, Jerusalem artichoke, and chicory	*Bifidobacterium* spp.	[85,86]
Galacto-oligosaccharide (GOS)	Fermentable	Enzymatic conversion of lactose, added in infant formula	*Bifidobacterium* spp. *Lactobacillus* spp.	[87]
β-glucan	Viscous, Fermentable	Bran of cereals such as oats and barley	*Bacteroides* spp. *Prevotella* spp. *Bifidobacterium* spp.	[88,89]
Pectin	Viscous, Fermentable	Pears, apples, berries, and oranges	*Bifidobacterium* spp. *Lactobacillus* spp. *Enterococcus* spp.	[90]
Gums (gum arabic)	Viscous, Fermentable	Substances that are secreted from plant cells in response to injury (gum arabic)	*Bifidobacterium* spp. *Lactobacillus* spp. *Bacteroides* spp.	[91]

## References

[1] Turnbaugh P.J., Ley R.E., Mahowald M.A., Magrini V., Mardis E.R., Gordon J.I. (2006). An Obesity-Associated Gut Microbiome with Increased Capacity for Energy Harvest. Nature.

[2] Forslund K., Hildebrand F., Nielsen T., Falony G., Le Chatelier E., Sunagawa S., Prifti E., Vieira-Silva S., Gudmundsdottir V., Krogh Pedersen H. (2015). Disentangling Type 2 Diabetes and Metformin Treatment Signatures in the Human Gut Microbiota. Nature.

[3] Vogt N.M., Kerby R.L., Dill-McFarland K.A., Harding S.J., Merluzzi A.P., Johnson S.C., Carlsson C.M., Asthana S., Zetterberg H., Blennow K. (2017). Gut Microbiome Alterations in Alzheimer’s Disease. Sci. Rep..

[4] Qiu C., Kivipelto M., von Strauss E. (2009). Epidemiology of Alzheimer’s Disease: Occurrence, Determinants, and Strategies toward Intervention. Dialogues Clin. Neurosci..

[5] Selkoe D.J. (2001). Alzheimer’s Disease: Genes, Proteins, and Therapy. Physiol. Rev..

[6] Mahley R.W., Weisgraber K.H., Huang Y. (2006). Apolipoprotein E4: A Causative Factor and Therapeutic Target in Neuropathology, Including Alzheimer’s Disease. Proc. Natl. Acad. Sci. USA.

[7] Strittmatter W.J., Saunders A.M., Schmechel D., Pericak-Vance M., Enghild J., Salvesen G.S., Roses A.D. (1993). Apolipoprotein E: High-Avidity Binding to Beta-Amyloid and Increased Frequency of Type 4 Allele in Late-Onset Familial Alzheimer Disease. Proc. Natl. Acad. Sci. USA.

[8] Baumgart M., Snyder H.M., Carrillo M.C., Fazio S., Kim H., Johns H. (2015). Summary of the Evidence on Modifiable Risk Factors for Cognitive Decline and Dementia: A Population-Based Perspective. Alzheimer’s Dement..

[9] Glenner G.G., Wong C.W. (1984). Alzheimer’s Disease: Initial Report of the Purification and Characterization of a Novel Cerebrovascular Amyloid Protein. Biochem. Biophys. Res. Commun..

[10] Wischik C.M., Novak M., Edwards P.C., Klug A., Tichelaar W., Crowther R.A. (1988). Structural Characterization of the Core of the Paired Helical Filament of Alzheimer Disease. Proc. Natl. Acad. Sci. USA.

[11] Sochocka M., Diniz B.S., Leszek J. (2017). Inflammatory Response in the CNS: Friend or Foe?. Mol. Neurobiol..

[12] Tohidpour A., Morgun A.V., Boitsova E.B., Malinovskaya N.A., Martynova G.P., Khilazheva E.D., Kopylevich N.V., Gertsog G.E., Salmina A.B. (2017). Neuroinflammation and Infection: Molecular Mechanisms Associated with Dysfunction of Neurovascular Unit. Front. Cell. Infect. Microbiol..

[13] Carabotti M., Scirocco A., Maselli M.A., Severi C. (2015). The Gut-Brain Axis: Interactions between Enteric Microbiota, Central and Enteric Nervous Systems. Ann. Gastroenterol..

[14] Bindels L.B., Delzenne N.M., Cani P.D., Walter J. (2015). Towards a More Comprehensive Concept for Prebiotics. Nat. Rev. Gastroenterol. Hepatol..

[15] Carlson J.L., Erickson J.M., Lloyd B.B., Slavin J.L. (2018). Health Effects and Sources of Prebiotic Dietary Fiber. Curr. Dev. Nutr..

[16] Filippo C.D., Cavalieri D., Paola M.D., Ramazzotti M., Poullet J.B., Massart S., Collini S., Pieraccini G., Lionetti P. (2010). Impact of Diet in Shaping Gut Microbiota Revealed by a Comparative Study in Children from Europe and Rural Africa. Proc. Natl. Acad. Sci. USA.

[17] Claesson M.J., Jeffery I.B., Conde S., Power S.E., O’Connor E.M., Cusack S., Harris H.M.B., Coakley M., Lakshminarayanan B., O’Sullivan O. (2012). Gut Microbiota Composition Correlates with Diet and Health in the Elderly. Nature.

[18] Kashyap P.C., Marcobal A., Ursell L.K., Larauche M., Duboc H., Earle K.A., Sonnenburg E.D., Ferreyra J.A., Higginbottom S.K., Million M. (2013). Complex Interactions Among Diet, Gastrointestinal Transit, and Gut Microbiota in Humanized Mice. Gastroenterology.

[19] Parks B.W., Nam E., Org E., Kostem E., Norheim F., Hui S.T., Pan C., Civelek M., Rau C.D., Bennett B.J. (2013). Genetic Control of Obesity and Gut Microbiota Composition in Response to High-Fat, High-Sucrose Diet in Mice. Cell Metab..

[20] Daniel H., Gholami A.M., Berry D., Desmarchelier C., Hahne H., Loh G., Mondot S., Lepage P., Rothballer M., Walker A. (2014). High-Fat Diet Alters Gut Microbiota Physiology in Mice. ISME J..

[21] Carmody R.N., Gerber G.K., Luevano J.M., Gatti D.M., Somes L., Svenson K.L., Turnbaugh P.J. (2015). Diet Dominates Host Genotype in Shaping the Murine Gut Microbiota. Cell Host Microbe.

[22] Filippis F.D., Pellegrini N., Vannini L., Jeffery I.B., Storia A.L., Laghi L., Serrazanetti D.I., Cagno R.D., Ferrocino I., Lazzi C. (2016). High-Level Adherence to a Mediterranean Diet Beneficially Impacts the Gut Microbiota and Associated Metabolome. Gut.

[23] Vaughn A.C., Cooper E.M., DiLorenzo P.M., O’Loughlin L.J., Konkel M.E., Peters J.H., Hajnal A., Sen T., Lee S.H., de La Serre C.B. (2017). Energy-Dense Diet Triggers Changes in Gut Microbiota, Reorganization of Gut-Brain Vagal Communication and Increases Body Fat Accumulation. Acta Neurobiol. Exp..

[24] Zou J., Chassaing B., Singh V., Pellizzon M., Ricci M., Fythe M.D., Kumar M.V., Gewirtz A.T. (2018). Fiber-Mediated Nourishment of Gut Microbiota Protects against Diet-Induced Obesity by Restoring IL-22-Mediated Colonic Health. Cell Host Microbe.

[25] Beilharz J.E., Kaakoush N.O., Maniam J., Morris M.J. (2018). Cafeteria Diet and Probiotic Therapy: Cross Talk among Memory, Neuroplasticity, Serotonin Receptors and Gut Microbiota in the Rat. Mol. Psychiatry.

[26] Hildebrandt M.A., Hoffmann C., Sherrill-Mix S.A., Keilbaugh S.A., Hamady M., Chen Y., Knight R., Ahima R.S., Bushman F., Wu G.D. (2009). High-Fat Diet Determines the Composition of the Murine Gut Microbiome Independently of Obesity. Gastroenterology.

[27] Walker A., Pfitzner B., Neschen S., Kahle M., Harir M., Lucio M., Moritz F., Tziotis D., Witting M., Rothballer M. (2014). Distinct Signatures of Host–Microbial Meta-Metabolome and Gut Microbiome in Two C57BL/6 Strains under High-Fat Diet. ISME J..

[28] Kim K.-A., Gu W., Lee I.-A., Joh E.-H., Kim D.-H. (2012). High Fat Diet-Induced Gut Microbiota Exacerbates Inflammation and Obesity in Mice via the TLR4 Signaling Pathway. PLoS ONE.

[29] de La Serre C.B., Ellis C.L., Lee J., Hartman A.L., Rutledge J.C., Raybould H.E. (2010). Propensity to High-Fat Diet-Induced Obesity in Rats Is Associated with Changes in the Gut Microbiota and Gut Inflammation. Am. J. Physiol.-Gastrointest. Liver Physiol..

[30] Lecomte V., Kaakoush N.O., Maloney C.A., Raipuria M., Huinao K.D., Mitchell H.M., Morris M.J. (2015). Changes in Gut Microbiota in Rats Fed a High Fat Diet Correlate with Obesity-Associated Metabolic Parameters. PLoS ONE.

[31] Kovatcheva-Datchary P., Nilsson A., Akrami R., Lee Y.S., De Vadder F., Arora T., Hallen A., Martens E., Björck I., Bäckhed F. (2015). Dietary Fiber-Induced Improvement in Glucose Metabolism Is Associated with Increased Abundance of Prevotella. Cell Metab..

[32] Wu G.D., Chen J., Hoffmann C., Bittinger K., Chen Y.-Y., Keilbaugh S.A., Bewtra M., Knights D., Walters W.A., Knight R. (2011). Linking Long-Term Dietary Patterns with Gut Microbial Enterotypes. Science.

[33] So D., Whelan K., Rossi M., Morrison M., Holtmann G., Kelly J.T., Shanahan E.R., Staudacher H.M., Campbell K.L. (2018). Dietary Fiber Intervention on Gut Microbiota Composition in Healthy Adults: A Systematic Review and Meta-Analysis. Am. J. Clin. Nutr..

[34] Bibbò S., Ianiro G., Giorgio V., Scaldaferri F., Masucci L., Gasbarrini A., Cammarota G. (2016). The Role of Diet on Gut Microbiota Composition. Eur. Rev. Med. Pharmacol. Sci..

[35] Jefferson A., Adolphus K. (2019). The Effects of Intact Cereal Grain Fibers, Including Wheat Bran on the Gut Microbiota Composition of Healthy Adults: A Systematic Review. Front. Nutr..

[36] Holscher H.D. (2017). Dietary Fiber and Prebiotics and the Gastrointestinal Microbiota. Gut Microbes.

[37] Ramirez-Farias C., Slezak K., Fuller Z., Duncan A., Holtrop G., Louis P. (2008). Effect of Inulin on the Human Gut Microbiota: Stimulation of Bifidobacterium Adolescentis and Faecalibacterium Prausnitzii. Br. J. Nutr..

[38] De Vuyst L., Leroy F. (2011). Cross-Feeding between Bifidobacteria and Butyrate-Producing Colon Bacteria Explains Bifdobacterial Competitiveness, Butyrate Production, and Gas Production. Int. J. Food Microbiol..

[39] Brandscheid C., Schuck F., Reinhardt S., Schäfer K.-H., Pietrzik C.U., Grimm M., Hartmann T., Schwiertz A., Endres K. (2017). Altered Gut Microbiome Composition and Tryptic Activity of the 5xFAD Alzheimer’s Mouse Model. J. Alzheimer’s Dis..

[40] Wu S.-C., Cao Z.-S., Chang K.-M., Juang J.-L. (2017). Intestinal Microbial Dysbiosis Aggravates the Progression of Alzheimer’s Disease in Drosophila. Nat. Commun..

[41] (2012). Human Microbiome Project Consortium Structure, Function and Diversity of the Healthy Human Microbiome. Nature.

[42] Sun B.-L., Li W.-W., Wang J., Xu Y.-L., Sun H.-L., Tian D.-Y., Wang Y.-J., Yao X.-Q. (2019). Gut Microbiota Alteration and Its Time Course in a Tauopathy Mouse Model. J. Alzheimer’s Dis..

[43] Bäuerl C., Collado M.C., Diaz Cuevas A., Viña J., Pérez Martínez G. (2018). Shifts in Gut Microbiota Composition in an APP/PSS1 Transgenic Mouse Model of Alzheimer’s Disease during Lifespan. Lett. Appl. Microbiol..

[44] Zhuang Z.-Q., Shen L.-L., Li W.-W., Fu X., Zeng F., Gui L., Lü Y., Cai M., Zhu C., Tan Y.-L. (2018). Gut Microbiota Is Altered in Patients with Alzheimer’s Disease. J. Alzheimer’s Dis..

[45] Wang X., Sun G., Feng T., Zhang J., Huang X., Wang T., Xie Z., Chu X., Yang J., Wang H. (2019). Sodium Oligomannate Therapeutically Remodels Gut Microbiota and Suppresses Gut Bacterial Amino Acids-Shaped Neuroinflammation to Inhibit Alzheimer’s Disease Progression. Cell Res..

[46] Crews L., Masliah E. (2010). Molecular Mechanisms of Neurodegeneration in Alzheimer’s Disease. Hum. Mol. Genet..

[47] Sochocka M., Donskow-Łysoniewska K., Diniz B.S., Kurpas D., Brzozowska E., Leszek J. (2019). The Gut Microbiome Alterations and Inflammation-Driven Pathogenesis of Alzheimer’s Disease—A Critical Review. Mol. Neurobiol..

[48] Cattaneo A., Cattane N., Galluzzi S., Provasi S., Lopizzo N., Festari C., Ferrari C., Guerra U.P., Paghera B., Muscio C. (2017). Association of Brain Amyloidosis with Pro-Inflammatory Gut Bacterial Taxa and Peripheral Inflammation Markers in Cognitively Impaired Elderly. Neurobiol. Aging.

[49] Hill E., Clifton P., Goodwill A.M., Dennerstein L., Campbell S., Szoeke C. (2018). Dietary Patterns and β-Amyloid Deposition in Aging Australian Women. Alzheimer’s Dement. Transl. Res. Clin. Interv..

[50] Nagpal R., Neth B.J., Wang S., Craft S., Yadav H. (2019). Modified Mediterranean-Ketogenic Diet Modulates Gut Microbiome and Short-Chain Fatty Acids in Association with Alzheimer’s Disease Markers in Subjects with Mild Cognitive Impairment. EBioMedicine.

[51] Neth B.J., Mintz A., Whitlow C., Jung Y., Solingapuram Sai K., Register T.C., Kellar D., Lockhart S.N., Hoscheidt S., Maldjian J. (2020). Modified Ketogenic Diet Is Associated with Improved Cerebrospinal Fluid Biomarker Profile, Cerebral Perfusion, and Cerebral Ketone Body Uptake in Older Adults at Risk for Alzheimer’s Disease: A Pilot Study. Neurobiol. Aging.

[52] Butterfield D.A., Di Domenico F., Barone E. (2014). Elevated Risk of Type 2 Diabetes for Development of Alzheimer Disease: A Key Role for Oxidative Stress in Brain. Biochim. Biophys. Acta (BBA) Mol. Basis Dis..

[53] Kubis-Kubiak A., Dyba A., Piwowar A. (2020). The Interplay between Diabetes and Alzheimer’s Disease-In the Hunt for Biomarkers. Int J Mol Sci.

[54] Chaudhuri J.R., Mridula K.R., Rathnakishore C., Anamika A., Samala N.R., Balaraju B., Bandaru V.S. (2020). Association Serum S100B Protein in Alzheimer’s Disease: A Case Control Study from South India. Curr. Alzheimer Res..

[55] Yu H., Li H., Liu X., Du X., Deng B. (2020). Levels of Serum S100B Are Associated with Cognitive Dysfunction in Patients with Type 2 Diabetes. Aging.

[56] Zhao L., Zhang F., Ding X., Wu G., Lam Y.Y., Wang X., Fu H., Xue X., Lu C., Ma J. (2018). Gut Bacteria Selectively Promoted by Dietary Fibers Alleviate Type 2 Diabetes. Science.

[57] Santos C.Y., Snyder P.J., Wu W.-C., Zhang M., Echeverria A., Alber J. (2017). Pathophysiologic Relationship between Alzheimer’s Disease, Cerebrovascular Disease, and Cardiovascular Risk: A Review and Synthesis. Alzheimers Dement..

[58] Tini G., Scagliola R., Monacelli F., La Malfa G., Porto I., Brunelli C., Rosa G.M. (2020). Alzheimer’s Disease and Cardiovascular Disease: A Particular Association. Cardiol. Res. Pract..

[59] Moran C., Phan T.G., Srikanth V.K. (2012). Cerebral Small Vessel Disease: A Review of Clinical, Radiological, and Histopathological Phenotypes. Int. J. Stroke.

[60] Thong J.Y.J., Hilal S., Wang Y., Soon H.W., Dong Y., Collinson S.L., Anh T.T., Ikram M.K., Wong T.Y., Venketasubramanian N. (2013). Association of Silent Lacunar Infarct with Brain Atrophy and Cognitive Impairment. J. Neurol. Neurosurg. Psychiatry.

[61] Love S., Miners J.S. (2016). Cerebrovascular Disease in Ageing and Alzheimer’s Disease. Acta Neuropathol..

[62] Deckers K., Schievink S.H.J., Rodriquez M.M.F., van Oostenbrugge R.J., van Boxtel M.P.J., Verhey F.R.J., Köhler S. (2017). Coronary Heart Disease and Risk for Cognitive Impairment or Dementia: Systematic Review and Meta-Analysis. PLoS ONE.

[63] Wolters F.J., Segufa R.A., Darweesh S.K.L., Bos D., Ikram M.A., Sabayan B., Hofman A., Sedaghat S. (2018). Coronary Heart Disease, Heart Failure, and the Risk of Dementia: A Systematic Review and Meta-Analysis. Alzheimer’s Dement..

[64] Stampfer M.J., Hu F.B., Manson J.E., Rimm E.B., Willett W.C. (2000). Primary Prevention of Coronary Heart Disease in Women through Diet and Lifestyle. N. Engl. J. Med..

[65] Rippe J.M. (2019). Lifestyle Strategies for Risk Factor Reduction, Prevention, and Treatment of Cardiovascular Disease. Am. J. Lifestyle Med..

[66] Kotseva K., De Backer G., De Bacquer D., Rydén L., Hoes A., Grobbee D., Maggioni A., Marques-Vidal P., Jennings C., Abreu A. (2019). Lifestyle and Impact on Cardiovascular Risk Factor Control in Coronary Patients across 27 Countries: Results from the European Society of Cardiology ESC-EORP EUROASPIRE V Registry. Eur. J. Prev. Cardiol..

[67] Ludwig D.S., Pereira M.A., Kroenke C.H., Hilner J.E., Horn L.V., Slattery M.L., David R., Jacobs J. (1999). Dietary Fiber, Weight Gain, and Cardiovascular Disease Risk Factors in Young Adults. JAMA.

[68] Mozaffarian D., Kumanyika S.K., Lemaitre R.N., Olson J.L., Burke G.L., Siscovick D.S. (2003). Cereal, Fruit, and Vegetable Fiber Intake and the Risk of Cardiovascular Disease in Elderly Individuals. JAMA.

[69] Lairon D., Arnault N., Bertrais S., Planells R., Clero E., Hercberg S., Boutron-Ruault M.-C. (2005). Dietary Fiber Intake and Risk Factors for Cardiovascular Disease in French Adults. Am. J. Clin. Nutr..

[70] Sahyoun N.R., Jacques P.F., Zhang X.L., Juan W., McKeown N.M. (2006). Whole-Grain Intake Is Inversely Associated with the Metabolic Syndrome and Mortality in Older Adults. Am. J. Clin. Nutr..

[71] Grooms K.N., Ommerborn M.J., Pham D.Q., Djoussé L., Clark C.R. (2013). Dietary Fiber Intake and Cardiometabolic Risks among US Adults, NHANES 1999-2010. Am. J. Med..

[72] Yan Q., Gu Y., Li X., Yang W., Jia L., Chen C., Han X., Huang Y., Zhao L., Li P. (2017). Alterations of the Gut Microbiome in Hypertension. Front. Cell. Infect. Microbiol..

[73] Kristensen M., Jensen M.G., Aarestrup J., Petersen K.E., Søndergaard L., Mikkelsen M.S., Astrup A. (2012). Flaxseed Dietary Fibers Lower Cholesterol and Increase Fecal Fat Excretion, but Magnitude of Effect Depend on Food Type. Nutr. Metab..

[74] Carvalho-Wells A.L., Helmolz K., Nodet C., Molzer C., Leonard C., McKevith B., Thielecke F., Jackson K.G., Tuohy K.M. (2010). Determination of the in Vivo Prebiotic Potential of a Maize-Based Whole Grain Breakfast Cereal: A Human Feeding Study. Br. J. Nutr..

[75] Prosky L., Asp N.G., Schweizer T.F., DeVries J.W., Furda I. (1988). Determination of Insoluble, Soluble, and Total Dietary Fiber in Foods and Food Products: Interlaboratory Study. J. Assoc. Off. Anal. Chem..

[76] Schneeman B.O. (1987). Soluble vs Insoluble Fiber: Different Physiological Responses. Food Technol..

[77] Pluta R., Ułamek-Kozioł M., Januszewski S., Czuczwar S.J. (2020). Gut Microbiota and pro/Prebiotics in Alzheimer’s Disease. Aging.

[78] Chassard C., Delmas E., Robert C., Bernalier-Donadille A. (2010). The Cellulose-Degrading Microbial Community of the Human Gut Varies According to the Presence or Absence of Methanogens. FEMS Microbiol. Ecol..

[79] Chassard C., Delmas E., Robert C., Lawson P.A., Bernalier-Donadille A. (2012). Ruminococcus Champanellensis Sp. Nov., a Cellulose-Degrading Bacterium from Human Gut Microbiota. Int. J. Syst. Evol. Microbiol..

[80] Robert C., Chassard C., Lawson P.A., Bernalier-Donadille A. (2007). Bacteroides Cellulosilyticus Sp. Nov., a Cellulolytic Bacterium from the Human Gut Microbial Community. Int. J. Syst. Evol. Microbiol..

[81] Wedekind K.J., Mansfield H.R., Montgomery L. (1988). Enumeration and Isolation of Cellulolytic and Hemicellulolytic Bacteria from Human Feces. Appl. Environ. Microbiol..

[82] Niemi P., Aura A.-M., Maukonen J., Smeds A.I., Mattila I., Niemelä K., Tamminen T., Faulds C.B., Buchert J., Poutanen K. (2013). Interactions of a Lignin-Rich Fraction from Brewer’s Spent Grain with Gut Microbiota in Vitro. J. Agric. Food Chem..

[83] Ze X., Duncan S.H., Louis P., Flint H.J. (2012). Ruminococcus Bromii Is a Keystone Species for the Degradation of Resistant Starch in the Human Colon. ISME J..

[84] Liu F., Li P., Chen M., Luo Y., Prabhakar M., Zheng H., He Y., Qi Q., Long H., Zhang Y. (2017). Fructooligosaccharide (FOS) and Galactooligosaccharide (GOS) Increase Bifidobacterium but Reduce Butyrate Producing Bacteria with Adverse Glycemic Metabolism in Healthy Young Population. Sci. Rep..

[85] Tarini J., Wolever T.M.S. (2010). The Fermentable Fibre Inulin Increases Postprandial Serum Short-Chain Fatty Acids and Reduces Free-Fatty Acids and Ghrelin in Healthy Subjects. Appl. Physiol. Nutr. Metab..

[86] Salazar N., Dewulf E.M., Neyrinck A.M., Bindels L.B., Cani P.D., Mahillon J., de Vos W.M., Thissen J.-P., Gueimonde M., de los Reyes-Gavilán C.G. (2015). Inulin-Type Fructans Modulate Intestinal Bifidobacterium Species Populations and Decrease Fecal Short-Chain Fatty Acids in Obese Women. Clin. Nutr..

[87] Cardelle-Cobas A., Corzo N., Olano A., Peláez C., Requena T., Ávila M. (2011). Galactooligosaccharides Derived from Lactose and Lactulose: Influence of Structure on Lactobacillus, Streptococcus and Bifidobacterium Growth. Int. J. Food Microbiol..

[88] Wang Y., Ames N.P., Tun H.M., Tosh S.M., Jones P.J., Khafipour E. (2016). High Molecular Weight Barley β-Glucan Alters Gut Microbiota Toward Reduced Cardiovascular Disease Risk. Front. Microbiol..

[89] Kristek A., Wiese M., Heuer P., Kosik O., Schär M.Y., Soycan G., Alsharif S., Kuhnle G.G.C., Walton G., Spencer J.P.E. (2019). Oat Bran, but Not Its Isolated Bioactive β-Glucans or Polyphenols, Have a Bifidogenic Effect in an in Vitro Fermentation Model of the Gut Microbiota. Br. J. Nutr..

[90] Shinohara K., Ohashi Y., Kawasumi K., Terada A., Fujisawa T. (2010). Effect of Apple Intake on Fecal Microbiota and Metabolites in Humans. Anaerobe.

[91] Calame W., Weseler A.R., Viebke C., Flynn C., Siemensma A.D. (2008). Gum Arabic Establishes Prebiotic Functionality in Healthy Human Volunteers in a Dose-Dependent Manner. Br. J. Nutr..

[92] Maier T.V., Lucio M., Lee L.H., VerBerkmoes N.C., Brislawn C.J., Bernhardt J., Lamendella R., McDermott J.E., Bergeron N., Heinzmann S.S. (2017). Impact of Dietary Resistant Starch on the Human Gut Microbiome, Metaproteome, and Metabolome. mBio.

[93] Biedrzycka E., Bielecka M. (2004). Prebiotic Effectiveness of Fructans of Different Degrees of Polymerization. Trends Food Sci. Technol..

[94] Moro G., Minoli I., Mosca M., Fanaro S., Jelinek J., Stahl B., Boehm G. (2002). Dosage-Related Bifidogenic Effects of Galacto- and Fructooligosaccharides in Formula-Fed Term Infants. J. Pediatric Gastroenterol. Nutr..

[95] Austin S., Bénet T., Michaud J., Cuany D., Rohfritsch P. Determination of β-Galactooligosaccharides by Liquid Chromatography. https://www.hindawi.com/journals/ijac/2014/768406/.

[96] Grabarics M., Csernák O., Balogh R., Béni S. (2017). Analytical Characterization of Human Milk Oligosaccharides—Potential Applications in Pharmaceutical Analysis. J. Pharm. Biomed. Anal..

[97] Zivkovic A., Lewis Z., German B., Mills D. (2013). Establishment of a Milk-Oriented-Microbiota (MOM) in Early Life: How Babies Meet Their MOMs. Food Rev. Int..

[98] Zivkovic A.M., German J.B., Lebrilla C.B., Mills D.A. (2011). Human Milk Glycobiome and Its Impact on the Infant Gastrointestinal Microbiota. Proc. Natl. Acad. Sci. USA.

[99] Kleessen B., Bunke H., Tovar K., Noack J., Sawatzki G. (1995). Influence of Two Infant Formulas and Human Milk on the Development of the Faecal Flora in Newborn Infants. Acta Paediatr..

[100] Martín R., Langa S., Reviriego C., Jimínez E., Marín M.L., Xaus J., Fernández L., Rodríguez J.M. (2003). Human Milk Is a Source of Lactic Acid Bacteria for the Infant Gut. J. Pediatrics.

[101] Marques T.M., Wall R., Ross R.P., Fitzgerald G.F., Ryan C.A., Stanton C. (2010). Programming Infant Gut Microbiota: Influence of Dietary and Environmental Factors. Curr. Opin. Biotechnol..

[102] Zhang Y., Wang P., Xia C., Wu Z., Zhong Z., Xu Y., Zeng Y., Liu H., Liu R., Liao M. (2020). Fructooligosaccharides Supplementation Mitigated Chronic Stress-Induced Intestinal Barrier Impairment and Neuroinflammation in Mice. J. Funct. Foods.

[103] Chen D., Yang X., Yang J., Lai G., Yong T., Tang X., Shuai O., Zhou G., Xie Y., Wu Q. (2017). Prebiotic Effect of Fructooligosaccharides from Morinda Officinalis on Alzheimer’s Disease in Rodent Models by Targeting the Microbiota-Gut-Brain Axis. Front. Aging Neurosci..

[104] Johansson L., Karesoja M., Ekholm P., Virkki L., Tenhu H. (2008). Comparison of the Solution Properties of (1→3),(1→4)-β-d-Glucans Extracted from Oats and Barley. LWT Food Sci. Technol..

[105] Regand A., Chowdhury Z., Tosh S.M., Wolever T.M.S., Wood P. (2011). The Molecular Weight, Solubility and Viscosity of Oat Beta-Glucan Affect Human Glycemic Response by Modifying Starch Digestibility. Food Chem..

[106] Lazaridou A., Biliaderis C.G. (2007). Molecular Aspects of Cereal β-Glucan Functionality: Physical Properties, Technological Applications and Physiological Effects. J. Cereal Sci..

[107] Arena M.P., Caggianiello G., Fiocco D., Russo P., Torelli M., Spano G., Capozzi V. (2014). Barley β-Glucans-Containing Food Enhances Probiotic Performances of Beneficial Bacteria. Int. J. Mol. Sci..

[108] Mohnen D. (2008). Pectin Structure and Biosynthesis. Curr. Opin. Plant Biol..

[109] Wei Y., Gong J., Zhu W., Tian H., Ding C., Gu L., Li N., Li J. (2016). Pectin Enhances the Effect of Fecal Microbiota Transplantation in Ulcerative Colitis by Delaying the Loss of Diversity of Gut Flora. BMC Microbiol..

[110] Churms S.C., Merrifield E.H., Stephen A.M. (1983). Some New Aspects of the Molecular Structure of Acacia Senegal Gum (Gum Arabic). Carbohydr. Res..

[111] Sanchez C., Schmitt C., Kolodziejczyk E., Lapp A., Gaillard C., Renard D. (2008). The Acacia Gum Arabinogalactan Fraction Is a Thin Oblate Ellipsoid: A New Model Based on Small-Angle Neutron Scattering and Ab Initio Calculation. Biophys. J..

[112] Roediger W.E. (1980). Role of Anaerobic Bacteria in the Metabolic Welfare of the Colonic Mucosa in Man. Gut.

[113] Cummings J.H., Pomare E.W., Branch W.J., Naylor C.P., Macfarlane G.T. (1987). Short Chain Fatty Acids in Human Large Intestine, Portal, Hepatic and Venous Blood. Gut.

[114] Kau A.L., Ahern P.P., Griffin N.W., Goodman A.L., Gordon J.I. (2011). Human Nutrition, the Gut Microbiome and the Immune System. Nature.

[115] Slavin J. (2013). Fiber and Prebiotics: Mechanisms and Health Benefits. Nutrients.

[116] Lupton J.R. (2004). Microbial Degradation Products Influence Colon Cancer Risk: The Butyrate Controversy. J. Nutr..

[117] van Limpt C., Crienen A., Vriesema A., Knol J. (2004). 134 Effect of Colonic Short Chain Fatty Acids, Lactate and PH on The Growth of Common Gut Pathogens. Pediatric Res..

[118] Parada Venegas D., De la Fuente M.K., Landskron G., González M.J., Quera R., Dijkstra G., Harmsen H.J.M., Faber K.N., Hermoso M.A. (2019). Short Chain Fatty Acids (SCFAs)-Mediated Gut Epithelial and Immune Regulation and Its Relevance for Inflammatory Bowel Diseases. Front. Immunol..

[119] Dass N.B., John A.K., Bassil A.K., Crumbley C.W., Shehee W.R., Maurio F.P., Moore G.B.T., Taylor C.M., Sanger G.J. (2007). The Relationship between the Effects of Short-Chain Fatty Acids on Intestinal Motility in Vitro and GPR43 Receptor Activation. Neurogastroenterol. Motil..

[120] Thangaraju M., Cresci G.A., Liu K., Ananth S., Gnanaprakasam J.P., Browning D.D., Mellinger J.D., Smith S.B., Digby G.J., Lambert N.A. (2009). GPR109A Is a G-Protein–Coupled Receptor for the Bacterial Fermentation Product Butyrate and Functions as a Tumor Suppressor in Colon. Cancer Res..

[121] Docampo M.D., Stein-Thoeringer C.K., Lazrak A., Burgos da Silva M.D., Cross J., van den Brink M.R.M. (2018). Expression of the Butyrate/Niacin Receptor, GPR109a on T Cells Plays an Important Role in a Mouse Model of Graft Versus Host Disease. Blood.

[122] Fu S.-P., Wang J.-F., Xue W.-J., Liu H.-M., Liu B., Zeng Y.-L., Li S.-N., Huang B.-X., Lv Q.-K., Wang W. (2015). Anti-Inflammatory Effects of BHBA in Both in Vivo and in Vitro Parkinson’s Disease Models Are Mediated by GPR109A-Dependent Mechanisms. J. Neuroinflammation.

[123] Singh N., Gurav A., Sivaprakasam S., Brady E., Padia R., Shi H., Thangaraju M., Prasad P.D., Manicassamy S., Munn D.H. (2014). Activation of Gpr109a, Receptor for Niacin and the Commensal Metabolite Butyrate, Suppresses Colonic Inflammation and Carcinogenesis. Immunity.

[124] Bellono N.W., Bayrer J.R., Leitch D.B., Castro J., Zhang C., O’Donnell T.A., Brierley S.M., Ingraham H.A., Julius D. (2017). Enterochromaffin Cells Are Gut Chemosensors That Couple to Sensory Neural Pathways. Cell.

[125] Chen Z., Luo J., Li J., Kim G., Stewart A., Urban J.F., Huang Y., Chen S., Wu L.-G., Chesler A. (2021). Interleukin-33 Promotes Serotonin Release from Enterochromaffin Cells for Intestinal Homeostasis. Immunity.

[126] Yano J.M., Yu K., Donaldson G.P., Shastri G.G., Ann P., Ma L., Nagler C.R., Ismagilov R.F., Mazmanian S.K., Hsiao E.Y. (2015). Indigenous Bacteria from the Gut Microbiota Regulate Host Serotonin Biosynthesis. Cell.

[127] Clarke G., Grenham S., Scully P., Fitzgerald P., Moloney R.D., Shanahan F., Dinan T.G., Cryan J.F. (2013). The Microbiome-Gut-Brain Axis during Early Life Regulates the Hippocampal Serotonergic System in a Sex-Dependent Manner. Mol. Psychiatry.

[128] Berger M., Gray J.A., Roth B.L. (2009). The Expanded Biology of Serotonin. Annu. Rev. Med..

[129] Brambilla P., Perez J., Barale F., Schettini G., Soares J.C. (2003). GABAergic Dysfunction in Mood Disorders. Mol. Psychiatry.

[130] Strandwitz P., Kim K.H., Terekhova D., Liu J.K., Sharma A., Levering J., McDonald D., Dietrich D., Ramadhar T.R., Lekbua A. (2019). GABA-Modulating Bacteria of the Human Gut Microbiota. Nat. Microbiol..

[131] Zheng P., Zeng B., Liu M., Chen J., Pan J., Han Y., Liu Y., Cheng K., Zhou C., Wang H. (2019). The Gut Microbiome from Patients with Schizophrenia Modulates the Glutamate-Glutamine-GABA Cycle and Schizophrenia-Relevant Behaviors in Mice. Sci. Adv..

[132] Strandwitz P. (2018). Neurotransmitter Modulation by the Gut Microbiota. Brain Res..

[133] Sherry C.L., Kim S.S., Dilger R.N., Bauer L.L., Moon M.L., Tapping R.I., Fahey G.C., Tappenden K.A., Freund G.G. (2010). Sickness Behavior Induced by Endotoxin Can Be Mitigated by the Dietary Soluble Fiber, Pectin, through up-Regulation of IL-4 and Th2 Polarization. Brain Behav. Immun..

[134] Matt S.M., Allen J.M., Lawson M.A., Mailing L.J., Woods J.A., Johnson R.W. (2018). Butyrate and Dietary Soluble Fiber Improve Neuroinflammation Associated With Aging in Mice. Front. Immunol..

[135] Yap Y.A., Mariño E. (2018). An Insight Into the Intestinal Web of Mucosal Immunity, Microbiota, and Diet in Inflammation. Front Immunol.

[136] Schroeder B.O., Birchenough G.M.H., Ståhlman M., Arike L., Johansson M.E.V., Hansson G.C., Bäckhed F. (2018). Bifidobacteria or Fiber Protects against Diet-Induced Microbiota-Mediated Colonic Mucus Deterioration. Cell Host Microbe.

[137] Desai M.S., Seekatz A.M., Koropatkin N.M., Kamada N., Hickey C.A., Wolter M., Pudlo N.A., Kitamoto S., Terrapon N., Muller A. (2016). A Dietary Fiber-Deprived Gut Microbiota Degrades the Colonic Mucus Barrier and Enhances Pathogen Susceptibility. Cell.

[138] Ruas-Madiedo P., Gueimonde M., Fernández-García M., de los Reyes-Gavilán C.G., Margolles A. (2008). Mucin Degradation by Bifidobacterium Strains Isolated from the Human Intestinal Microbiota. Appl. Environ. Microbiol..

[139] Yoshihara T., Oikawa Y., Kato T., Kessoku T., Kobayashi T., Kato S., Misawa N., Ashikari K., Fuyuki A., Ohkubo H. (2020). The Protective Effect of Bifidobacterium Bifidum G9-1 against Mucus Degradation by Akkermansia Muciniphila Following Small Intestine Injury Caused by a Proton Pump Inhibitor and Aspirin. Gut Microbes.

[140] Ottman N., Geerlings S.Y., Aalvink S., de Vos W.M., Belzer C. (2017). Action and Function of Akkermansia Muciniphila in Microbiome Ecology, Health and Disease. Best Pract. Res. Clin. Gastroenterol..

[141] Plovier H., Everard A., Druart C., Depommier C., Van Hul M., Geurts L., Chilloux J., Ottman N., Duparc T., Lichtenstein L. (2017). A Purified Membrane Protein from Akkermansia Muciniphila or the Pasteurized Bacterium Improves Metabolism in Obese and Diabetic Mice. Nat. Med..

[142] Depommier C., Everard A., Druart C., Plovier H., Van Hul M., Vieira-Silva S., Falony G., Raes J., Maiter D., Delzenne N.M. (2019). Supplementation with Akkermansia Muciniphila in Overweight and Obese Human Volunteers: A Proof-of-Concept Exploratory Study. Nat. Med..

[143] Xu Y., Wang N., Tan H.-Y., Li S., Zhang C., Feng Y. (2020). Function of Akkermansia Muciniphila in Obesity: Interactions With Lipid Metabolism, Immune Response and Gut Systems. Front. Microbiol..

[144] Heintz-Buschart A., Pandey U., Wicke T., Sixel-Döring F., Janzen A., Sittig-Wiegand E., Trenkwalder C., Oertel W.H., Mollenhauer B., Wilmes P. (2018). The Nasal and Gut Microbiome in Parkinson’s Disease and Idiopathic Rapid Eye Movement Sleep Behavior Disorder. Mov. Disord..

[145] Cani P.D. (2018). Human Gut Microbiome: Hopes, Threats and Promises. Gut.

[146] Peng L., He Z., Chen W., Holzman I.R., Lin J. (2007). Effects of Butyrate on Intestinal Barrier Function in a Caco-2 Cell Monolayer Model of Intestinal Barrier. Pediatric Res..

[147] Wang H.-B., Wang P.-Y., Wang X., Wan Y.-L., Liu Y.-C. (2012). Butyrate Enhances Intestinal Epithelial Barrier Function via Up-Regulation of Tight Junction Protein Claudin-1 Transcription. Dig. Dis. Sci..

[148] Peng L., Li Z.-R., Green R.S., Holzman I.R., Lin J. (2009). Butyrate Enhances the Intestinal Barrier by Facilitating Tight Junction Assembly via Activation of AMP-Activated Protein Kinase in Caco-2 Cell Monolayers. J. Nutr..

[149] Bourassa M.W., Alim I., Bultman S.J., Ratan R.R. (2016). Butyrate, Neuroepigenetics and the Gut Microbiome: Can a High Fiber Diet Improve Brain Health?. Neurosci. Lett..

[150] Friedland R.P., Budinger T.F., Ganz E., Yano Y., Mathis C.A., Koss B., Ober B.A., Huesman R.H., Derenzo S.E. (1983). Regional Cerebral Metabolic Alterations in Dementia of the Alzheimer Type: Positron Emission Tomography with [18F]Fluorodeoxyglucose. J. Comput. Assist. Tomogr..

[151] Mosconi L., Pupi A., De Leon M.J. (2008). Brain Glucose Hypometabolism and Oxidative Stress in Preclinical Alzheimer’s Disease. Ann. N. Y. Acad. Sci..

[152] Shimada Y., Kinoshita M., Harada K., Mizutani M., Masahata K., Kayama H., Takeda K. (2013). Commensal Bacteria-Dependent Indole Production Enhances Epithelial Barrier Function in the Colon. PLoS ONE.

[153] Venkatesh M., Mukherjee S., Wang H., Li H., Sun K., Benechet A.P., Qiu Z., Maher L., Redinbo M.R., Phillips R.S. (2014). Symbiotic Bacterial Metabolites Regulate Gastrointestinal Barrier Function via the Xenobiotic Sensor PXR and Toll-like Receptor 4. Immunity.

[154] Li J., Zhang L., Wu T., Li Y., Zhou X., Ruan Z. (2021). Indole-3-Propionic Acid Improved the Intestinal Barrier by Enhancing Epithelial Barrier and Mucus Barrier. J. Agric. Food Chem..

[155] Gheorghe C.E., Martin J.A., Manriquez F.V., Dinan T.G., Cryan J.F., Clarke G. (2019). Focus on the Essentials: Tryptophan Metabolism and the Microbiome-Gut-Brain Axis. Curr. Opin. Pharmacol..

[156] Pappolla M.A., Perry G., Fang X., Zagorski M., Sambamurti K., Poeggeler B. (2021). Indoles as Essential Mediators in the Gut-Brain Axis. Their Role in Alzheimer’s Disease. Neurobiol. Dis..

[157] Hwang I.K., Yoo K.-Y., Li H., Park O.K., Lee C.H., Choi J.H., Jeong Y.-G., Lee Y.L., Kim Y.-M., Kwon Y.-G. (2009). Indole-3-Propionic Acid Attenuates Neuronal Damage and Oxidative Stress in the Ischemic Hippocampus. J. Neurosci. Res..

[158] Bozner P., Grishko V., LeDoux S.P., Wilson G.L., Chyan Y.-C., Pappolla M.A. (1997). The Amyloid β Protein Induces Oxidative Damage of Mitochondrial DNA. J. Neuropathol. Exp. Neurol..

[159] Poeggeler B., Sambamurti K., Siedlak S.L., Perry G., Smith M.A., Pappolla M.A. (2010). A Novel Endogenous Indole Protects Rodent Mitochondria and Extends Rotifer Lifespan. PLoS ONE.

[160] Jaglin M., Rhimi M., Philippe C., Pons N., Bruneau A., Goustard B., Daugé V., Maguin E., Naudon L., Rabot S. (2018). Indole, a Signaling Molecule Produced by the Gut Microbiota, Negatively Impacts Emotional Behaviors in Rats. Front. Neurosci..

[161] Ohtsuki S., Asaba H., Takanaga H., Deguchi T., Hosoya K., Otagiri M., Terasaki T. (2002). Role of Blood–Brain Barrier Organic Anion Transporter 3 (OAT3) in the Efflux of Indoxyl Sulfate, a Uremic Toxin: Its Involvement in Neurotransmitter Metabolite Clearance from the Brain. J. Neurochem..

[162] Watanabe K., Watanabe T., Nakayama M. (2014). Cerebro-Renal Interactions: Impact of Uremic Toxins on Cognitive Function. NeuroToxicology.

[163] Molinero N., Ruiz L., Sánchez B., Margolles A., Delgado S. (2019). Intestinal Bacteria Interplay with Bile and Cholesterol Metabolism: Implications on Host Physiology. Front. Physiol..

[164] Kim I., Ahn S.-H., Inagaki T., Choi M., Ito S., Guo G.L., Kliewer S.A., Gonzalez F.J. (2007). Differential Regulation of Bile Acid Homeostasis by the Farnesoid X Receptor in Liver and Intestine. J. Lipid Res..

[165] MahmoudianDehkordi S., Arnold M., Nho K., Ahmad S., Jia W., Xie G., Louie G., Kueider-Paisley A., Moseley M.A., Thompson J.W. (2019). Altered Bile Acid Profile Associates with Cognitive Impairment in Alzheimer’s Disease—An Emerging Role for Gut Microbiome. Alzheimer’s Dement..

[166] Bennett D.A., Buchman A.S., Boyle P.A., Barnes L.L., Wilson R.S., Schneider J.A. (2018). Religious Orders Study and Rush Memory and Aging Project. J. Alzheimers Dis..

[167] Zhu C., Sawrey-Kubicek L., Beals E., Rhodes C.H., Houts H.E., Sacchi R., Zivkovic A.M. (2020). Human Gut Microbiome Composition and Tryptophan Metabolites Were Changed Differently by Fast Food and Mediterranean Diet in 4 Days: A Pilot Study. Nutr. Res..

[168] Hebert J.C., Radford-Smith D.E., Probert F., Ilott N., Chan K.W., Anthony D.C., Burnet P.W.J. (2021). Mom’s Diet Matters: Maternal Prebiotic Intake in Mice Reduces Anxiety and Alters Brain Gene Expression and the Fecal Microbiome in Offspring. Brain Behav. Immun..

[169] Burokas A., Arboleya S., Moloney R.D., Peterson V.L., Murphy K., Clarke G., Stanton C., Dinan T.G., Cryan J.F. (2017). Targeting the Microbiota-Gut-Brain Axis: Prebiotics Have Anxiolytic and Antidepressant-like Effects and Reverse the Impact of Chronic Stress in Mice. Biol. Psychiatry.

[170] Chunchai T., Thunapong W., Yasom S., Wanchai K., Eaimworawuthikul S., Metzler G., Thiennimitr P., Sirilun S., Chaiyasut C., Lungkaphin A. (2017). PREBIOTICS, PROBIOTICS OR SYNBIOTICS THERAPY RESTORES COGNITIVE DECLINE IN OBESE RATS. Alzheimer’s Dement..

[171] Liu Q., Xi Y., Wang Q., Liu J., Li P., Meng X., Liu K., Chen W., Liu X., Liu Z. (2021). Mannan Oligosaccharide Attenuates Cognitive and Behavioral Disorders in the 5xFAD Alzheimer’s Disease Mouse Model via Regulating the Gut Microbiota-Brain Axis. Brain Behav. Immun..

[172] Grimaldi R., Gibson G.R., Vulevic J., Giallourou N., Castro-Mejía J.L., Hansen L.H., Leigh Gibson E., Nielsen D.S., Costabile A. (2018). A Prebiotic Intervention Study in Children with Autism Spectrum Disorders (ASDs). Microbiome.

[173] Silk D.B.A., Davis A., Vulevic J., Tzortzis G., Gibson G.R. (2009). Clinical Trial: The Effects of a Trans-Galactooligosaccharide Prebiotic on Faecal Microbiota and Symptoms in Irritable Bowel Syndrome. Aliment. Pharmacol. Ther..

[174] Azpiroz F., Dubray C., Bernalier-Donadille A., Cardot J.-M., Accarino A., Serra J., Wagner A., Respondek F., Dapoigny M. (2017). Effects of ScFOS on the Composition of Fecal Microbiota and Anxiety in Patients with Irritable Bowel Syndrome: A Randomized, Double Blind, Placebo Controlled Study. Neurogastroenterol. Motil..

[175] Smith A.P., Sutherland D., Hewlett P. (2015). An Investigation of the Acute Effects of Oligofructose-Enriched Inulin on Subjective Wellbeing, Mood and Cognitive Performance. Nutrients.

[176] Leyrolle Q., Cserjesi R., Mulders M.D., Zamariola G., Hiel S., Gianfrancesco M.A., Portheault D., Amadieu C., Bindels L.B., Leclercq S. (2021). Prebiotic Effect on Mood in Obese Patients Is Determined by the Initial Gut Microbiota Composition: A Randomized, Controlled Trial. Brain Behav. Immun..

[177] Gibson G.R., Beatty E.R., Wang X., Cummings J.H. (1995). Selective Stimulation of Bifidobacteria in the Human Colon by Oligofructose and Inulin. Gastroenterology.

[178] Savignac H.M., Kiely B., Dinan T.G., Cryan J.F. (2014). Bifidobacteria Exert Strain-Specific Effects on Stress-Related Behavior and Physiology in BALB/c Mice. Neurogastroenterol. Motil..

[179] Savignac H.M., Tramullas M., Kiely B., Dinan T.G., Cryan J.F. (2015). Bifidobacteria Modulate Cognitive Processes in an Anxious Mouse Strain. Behav. Brain Res..

[180] Allen A.P., Hutch W., Borre Y.E., Kennedy P.J., Temko A., Boylan G., Murphy E., Cryan J.F., Dinan T.G., Clarke G. (2016). Bifidobacterium Longum 1714 as a Translational Psychobiotic: Modulation of Stress, Electrophysiology and Neurocognition in Healthy Volunteers. Transl. Psychiatry.

[181] Talbott S., Talbott J. (2009). Effect of BETA 1, 3/1, 6 GLUCAN on Upper Respiratory Tract Infection Symptoms and Mood State in Marathon Athletes. J. Sports Sci. Med..

[182] Talbott S.M., Talbott J.A. (2012). Baker’s Yeast Beta-Glucan Supplement Reduces Upper Respiratory Symptoms and Improves Mood State in Stressed Women. J. Am. Coll. Nutr..

[183] Best T., Kemps E., Bryan J. (2009). Saccharide Effects on Cognition and Well-Being in Middle-Aged Adults: A Randomized Controlled Trial. Dev. Neuropsychol..

[184] Best T., Howe P., Bryan J., Buckley J., Scholey A. (2015). Acute Effects of a Dietary Non-Starch Polysaccharide Supplement on Cognitive Performance in Healthy Middle-Aged Adults. Nutr. Neurosci..

[185] Berding K., Long-Smith C.M., Carbia C., Bastiaanssen T.F.S., van de Wouw M., Wiley N., Strain C.R., Fouhy F., Stanton C., Cryan J.F. (2021). A Specific Dietary Fibre Supplementation Improves Cognitive Performance-an Exploratory Randomised, Placebo-Controlled, Crossover Study. Psychopharmacology.

[186] Prasad S., Dhiman R.K., Duseja A., Chawla Y.K., Sharma A., Agarwal R. (2007). Lactulose Improves Cognitive Functions and Health-Related Quality of Life in Patients with Cirrhosis Who Have Minimal Hepatic Encephalopathy. Hepatology.

